# AFCF-Net: A novel U-Net based asymmetric feature calibration and fusion network for skin lesion image segmentation

**DOI:** 10.1371/journal.pone.0314000

**Published:** 2024-11-25

**Authors:** Zhanlin Ji, Zidong Yu, Chunling Liu, Zhiwu Wang, Shengnan Hao, Ivan Ganchev

**Affiliations:** 1 College of Mathematics and Computer Science, Zhejiang A&F University, Hangzhou, China; 2 Telecommunications Research Centre (TRC), University of Limerick, Limerick, Ireland; 3 Hebei Key Laboratory of Industrial Intelligent Perception, North China University of Science and Technology, Tangshan, Hebei, China; 4 Department of Chemoradiotherapy, Tangshan People’s Hospital, Tangshan, Hebei, China; 5 Department of Computer Systems, University of Plovdiv “Paisii Hilendarski”, Plovdiv, Bulgaria; 6 Institute of Mathematics and Informatics—Bulgarian Academy of Sciences, Sofia, Bulgaria; Wuhan University of Science and Technology, CHINA

## Abstract

Skin lesion segmentation plays a pivotal role in the diagnosis and treatment of skin diseases. By using deep neural networks to segment lesion areas, doctors can more accurately assess the severity of health-related conditions of patients and promptly implement appropriate treatment measures, thereby enhancing treatment outcomes and improving the quality of life (QoL) of patients. However, existing segmentation networks still face challenges in balancing segmentation performance and efficiency. To address this issue, a novel network, named AFCF-Net, is proposed in this paper for skin lesion segmentation tasks. Firstly, the proposed network employs a newly designed spatial channel feature calibration convolution (SCFCConv) to enhance its ability to perceive spatial and channel features. Secondly, AFCF-Net utilizes newly designed feature symmetric fusion convolution (FSFConv) in skip connections to selectively fuse features from different levels, thereby enhancing its sensitivity to texture, edges, and other detailed features. In addition, a feature attention recombination module (FARM) is added to the bottleneck of the proposed network to comprehensively acquire and utilize contextual information at different scales, thus improving the network’s generalization ability. Finally, a newly designed multi-level feature aggregation branch is introduced as an additional decoder for AFCF-Net to supplement key features lost during the original decoding process. Experiments, conducted on four skin image datasets, demonstrate that the proposed AFCF-Net network achieves better segmentation performance with fewer parameters and computational resources, compared to state-of-the-art segmentation networks. Additionally, AFCF-Net exhibits stronger generalization ability.

## 1 Introduction

Skin cancer is caused by malignant tumors formed by the abnormal proliferation of skin cells, with lethality and rapid spread, making it one of the prevalent cancers today [1]. According to the World Health Organization (WHO) survey, skin cancer accounts for one-third of diagnosed cancer cases, and its incidence rate continues to rise annually [2]. Skin cancer is classified into various types, among which melanoma is the most severe one, with an extremely high mortality rate, making it one of the main causes of death among skin cancer patients [3]. Global statistics indicate approximately 132,000 diagnosed cases of melanoma each year, resulting in an average of more than two deaths from this disease every hour [4]. However, melanoma is not incurable; early diagnosis can significantly improve patient survival rates. After professional treatment, the survival rate of early cases can exceed 90% [5]. In the diagnosis of skin diseases, dermoscopic images and clinical images are the two most commonly used imaging modalities [6]. However, even with the assistance of these two imaging modalities for dermatologists, diagnostic discrepancies may still occur due to certain unavoidable factors (such as the subjective judgment of clinical doctors, poor image quality, etc.), thereby affecting diagnostic accuracy. Therefore, there is an urgent need for efficient automated skin lesion segmentation techniques to minimize differences in diagnostic outcomes and improve the accuracy and efficiency of skin disease diagnosis.

In recent years, convolutional neural networks (CNNs) [7] have demonstrated immense potential as a powerful deep learning technique in the field of computer vision. By automatically learning the features of lesions in images, CNN s can accurately and efficiently segment lesions, thereby reducing the workload and subjective errors associated with manual segmentation performed by doctors. Initially, Long et al. [8] introduced the deconvolution operation into traditional CNNs, designing the Fully Convolutional Network (FCN). However, due to the small-sample nature of medical image datasets, insufficient training was provided to FCN during its training, which limits the network’s ability to judge lesion details and low-contrast areas [9]. Subsequently, Ronneberger et al. proposed the U-Net network [10], which consists of symmetric encoder and decoder structures, and passes features between them via skip connections, thereby capable of handling feature information at different scales. Due to its excellent segmentation performance in small-sample segmentation tasks, this network has been widely applied in medical image segmentation tasks. In [11], Zhou et al. investigated the optimal depth of the network and proposed UNet++. They introduced dense connection operations into the skip connections of U-Net to compensate for semantic differences between feature maps at different levels. However, this makes the network structure more complex, significantly increasing the computational burden. Recently, some networks [12–14] have incorporated self-attention mechanisms, endowing them with more powerful feature extraction capabilities, thereby improving their overall segmentation performance. However, the current challenge is how to balance the network’s segmentation accuracy and efficiency for skin disease images, enabling a network to achieve higher segmentation precision and stronger generalization ability with lower resource consumption. Some networks pursue higher segmentation performance at the expense of computational efficiency, while others focus more on segmentation efficiency but perform poorly in segmentation tasks with low generalization ability. Therefore, further research is needed to explore new methods to achieve balance between segmentation performance and efficiency, thus better meeting the requirements of skin lesion segmentation tasks [15].

To address the aforementioned issues, this paper proposes a novel network, called AFCF-Net, based on U-Net. AFCF-Net adopts an asymmetric encoder-decoder structure, which allows it to achieve state-of-the-art level in both segmentation performance and efficiency, while ensuring low computational and parameter overhead, which is demonstrated through experiments conducted on four datasets.

The main contributions of this paper can be summarized as follows:

1. A newly designed spatial channel feature calibration convolution (SCFCConv) is elaborated for use in the proposed AFCF-Net network as to significantly enhance its feature extraction capability by validating and integrating spatial feature information and channel relationships, while effectively reducing redundancy among features.
2. A novel feature symmetric fusion convolution (FSFConv) is developed for utilization in a new skip connection utilized by the proposed AFCF-Net network. FSFConv distinguishes features of different frequencies within the encoder/decoder by emphasizing high-frequency features, thus enabling the network to better capture the textures and edge features of skin lesions.
3. A newly designed feature attention recombination module (FARM) is proposed for use in AFCF-Net in order to extract and integrate multi-scale features, providing the decoder with a richer and more comprehensive feature representation.
4. A newly designed multi-level feature aggregation branch is integrated into the proposed network as a second decoder, which is able to enhance and fuse critical features from multiple levels of the original decoder, providing additional information support to improve the network’s generalization ability.

## 2 Related works

### 2.1 Skin lesion segmentation

Deep learning techniques have made considerable progress in various fields, especially in image processing and computer vision. In recent years, various deep networks have demonstrated outstanding performance in medical image segmentation tasks. In 2015, Ronneberger et al. [10] proposed the U-Net network, which was specifically designed for biomedical image segmentation. This network significantly improved image segmentation on small datasets by introducing a symmetric encoder-decoder structure and skip connections, thus becoming an important benchmark network in the field. Later, to reduce the parameter burden of U-Net, Valanarasu et al. [16] proposed the UNeXt network, based on convolutional multilayer perceptrons. The network effectively labels and projects convolutional features using tokenized multilayer perceptron blocks. Subsequently, Ruan et al. [17] designed the lightweight skin lesion segmentation network MALUNet, based on U-Net. They combined dilated convolutions with gated attention mechanisms to design a DGA block for extracting global and local feature information, and used an IEA block to enhance inter-sample relationships. Additionally, they incorporated SAB and CAB blocks into the network’s skip connections to generate attention maps from spatial and channel dimensions, achieving a more lightweight medical image segmentation. In recent years, the Visual Transformer (ViT) have been widely used in the field of image segmentation. Ruan et al. [18] designed the Multi-axis External Weight UNet (MEW-UNet), based on a U-shape architecture. They improved the network’s segmentation performance by replacing the traditional self-attention in ViT with newly designed Multi-axis External Weight (MEW) blocks, effectively utilizing frequency domain information. Recently, the Mamba architecture has garnered attention from researchers due to its advantages in long-range modeling. In 2024, Ruan et al. [19] combined U-Net with Mamba to design the Vision Mamba UNet (VM-UNet), which captures broader contextual information by introducing the Visual Spatial (VSS) block. VM-UNet has been validated on multiple datasets.

### 2.2 Attention mechanisms

Inspired by humans’ ability to focus on salient objects in complex scenes, networks combining attention mechanisms with deep learning have attracted widespread attention in the field of medical image analysis. In the field of computer vision, attention is a mechanism that selectively allows a network to focus on key information in input feature maps. In 2014, Mnih et al. [20] integrated neural networks with attention mechanisms for the first time, employing attention mechanisms on recurrent neural network (RNN) to iteratively predict important regions and update the entire network. Later, Hu et al. [21] proposed a novel channel attention module, called SENet, introduced global average pooling and fully connected layers to capture image dimension information and emphasized the feature representation of important channels by suppressing the noise. Subsequently, Woo et al. [22] proposed the CBAM attention module, which can simultaneously focus on features in both spatial and channel dimensions, enhancing computational efficiency by decoupling the channel attention from the spatial attention. To alleviate the computational complexity of networks, Wang et al. [23] proposed a lightweight Efficient Channel Attention (ECA) mechanism, which achieved efficient local cross-channel interaction through one-dimensional convolution. Moreover, since it only considers the relationships between each channel and its neighboring channels, it can fully extract feature dependencies between channels while maintaining a lower computational complexity. Later, various attention mechanisms emerged aimed at reducing the negative impact of noise on network performance.

In this paper, we combine the ECA attention [23] with feature fusion and a newly designed multi-level feature aggregation branch to enhance and fuse key information in the original U-Net decoder, thus comprehensively utilizing multi-scale features by it, allowing to improve the generalization ability of the proposed network.

### 2.3 Loss functions

During the training process of deep networks, the loss function plays a crucial role in measuring the difference between the network’s predicted output and the ground-truth labels. Hence, the selection of the loss function directly influences both the training efficacy and ultimate performance of a network.

The Binary Cross-Entropy (BCE) loss [24] is widely used in binary classification tasks. Compared to other loss functions, the BCE loss penalizes prediction errors more prominently, making the network less susceptible to noise [25]. However, in the task of skin lesion segmentation, the frequency of appearance of different types of skin lesions in the images is uneven. In such cases, using the BCE loss function would lead the network to focus more on classes with higher frequencies of appearance while neglecting those with lower frequencies. Additionally, the BCE loss usually only considers the classification accuracy at the pixel level and ignores the spatial correlation between pixels. This can lead to a network not being able to make full use of the spatial correlation information, which in turn may result in insufficiently smooth boundaries and fragmentation of the segmentation results [26].

To address the aforementioned issues, in this paper, we ingeniously combine the BCE loss [24] with the Dice loss [27] for use with the proposed AFCF-Net network. The Dice loss function can fully consider the spatial similarity between pixels, helping the network utilize spatial dependency information between pixels. Combining these two loss functions allows the network to comprehensively consider classification accuracy and segmentation boundary smoothness, thereby improving its overall performance in skin lesion segmentation tasks. The combined BCE-Dice loss is calculated as follows:

$$L_{B}=-\frac{1}{N}\sum_{i=1}^{N}\left(t_{i}\;\log\;p_{i}+(1-t_{i})\log\left(1-p_{i}\right)\right)$$ (1)

$$L_{D}=1-\frac{2\sum_{i=1}^{N}p_{i}t_{i}}{\sum_{i=1}^{N}p_{i}^{2}+\sum_{i=1}^{N}t_{i}^{2}}$$ (2)

$$L=L_{D}+\frac{1}{2}L_{B}$$ (3)

where *p*_*i*_ denotes the values predicted by the network, *t*_*i*_ denotes the true-label values, and *N* denotes the total number of pixels.

## 3 Method

### 3.1 AFCF-Net overall structure

**Fig 1:**
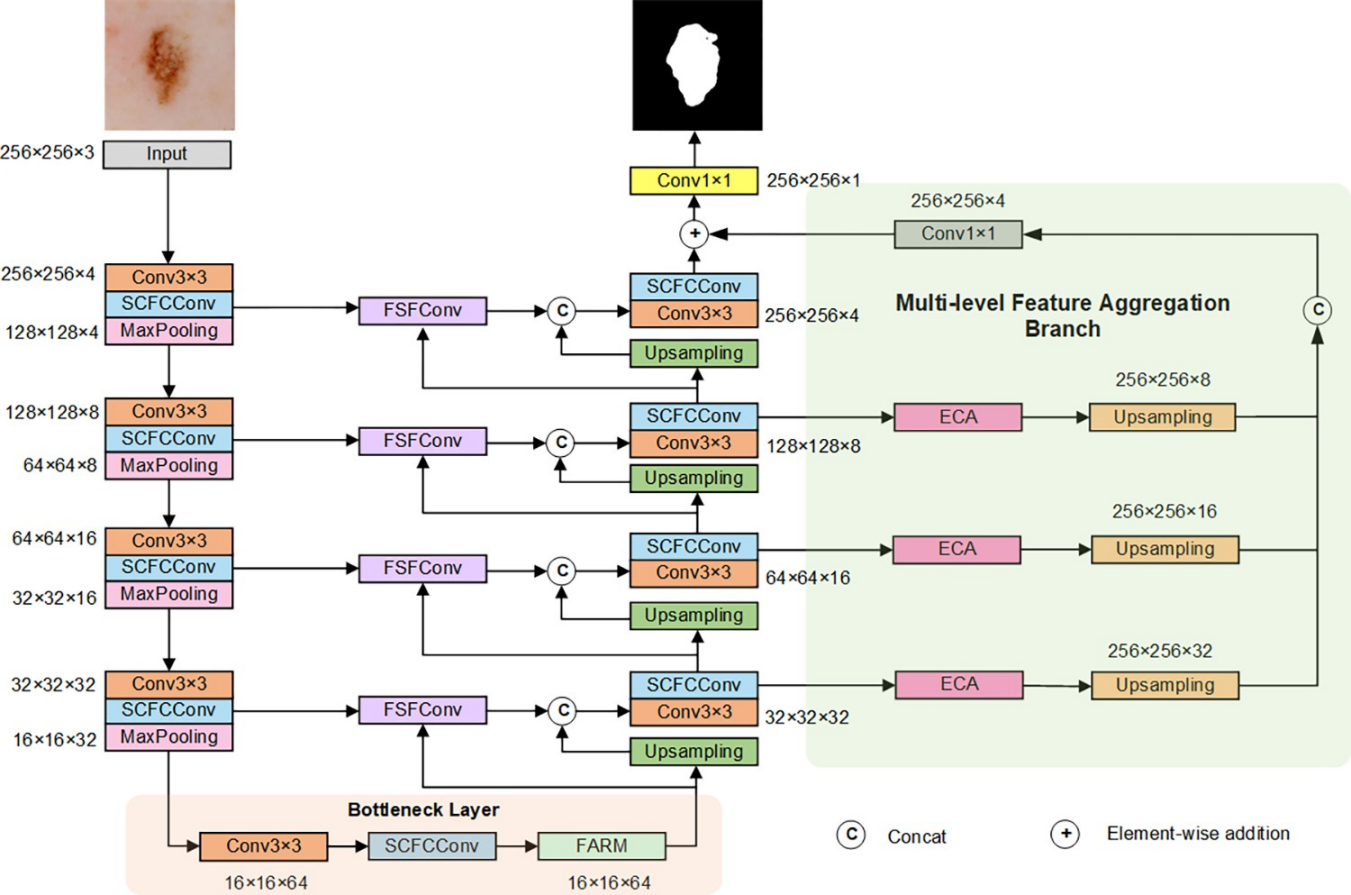
The overall structure of the proposed AFCF-Net network.

This paper proposes a novel U-shaped network, called AFCF-Net, with an asymmetric encoder-decoder structure, depicted in Fig 1, which is based on the U-Net network. Because in skin lesion image segmentation tasks the performance of a network largely depends on its ability to acquire spatial features, multi-scale contextual information, and boundary texture features, several newly designed modules are integrated into AFCF-Net to improve its performance in skin lesion segmentation tasks. These modules are detailed in the following subsections.

The proposed AFCF-Net network consists of five main parts: encoder, decoder, bottleneck, skip connections, and a multi-level feature aggregation branch. After inputting the skin lesion images into the network, they undergo encoding of lesion features through four layers. The AFCF-Net encoder utilizes a combination of 3×3 convolutions, newly elaborated SCFCConv, and max pooling to endow the network with the ability to localize and capture diverse lesion features in the images. At the bottleneck of the network, a newly designed FARM is employed to extract rich multi-scale contextual information from the encoder’s output features, enabling the network to more effectively discern the relationship between lesion targets and background in the images. In the skip connections of AFCF-Net, newly elaborated type of convolution, named FSFConv, is used to selectively fuse feature information from the encoder and decoder prior to upsampling, highlighting boundary textures and detailed features, aiding the network in better understanding the anisotropy of lesion features. Subsequently, the detailed lesion features are restored through four layers of the decoder, configured similarly to the encoder. Additionally, through the newly designed multi-level feature aggregation branch, information at multiple layers of the decoder is enhanced and fused, enabling the network to utilize multi-level features more effectively, thereby improving its ability to recognize lesion areas. By incorporating these advanced modules, AFCF-Net achieves more accurate skin lesion segmentation compared to state-of-the-art networks, while also significantly reducing the number of its parameters and computational complexity.

### 3.2 Spatial channel feature calibration convolution

**Fig 2:**
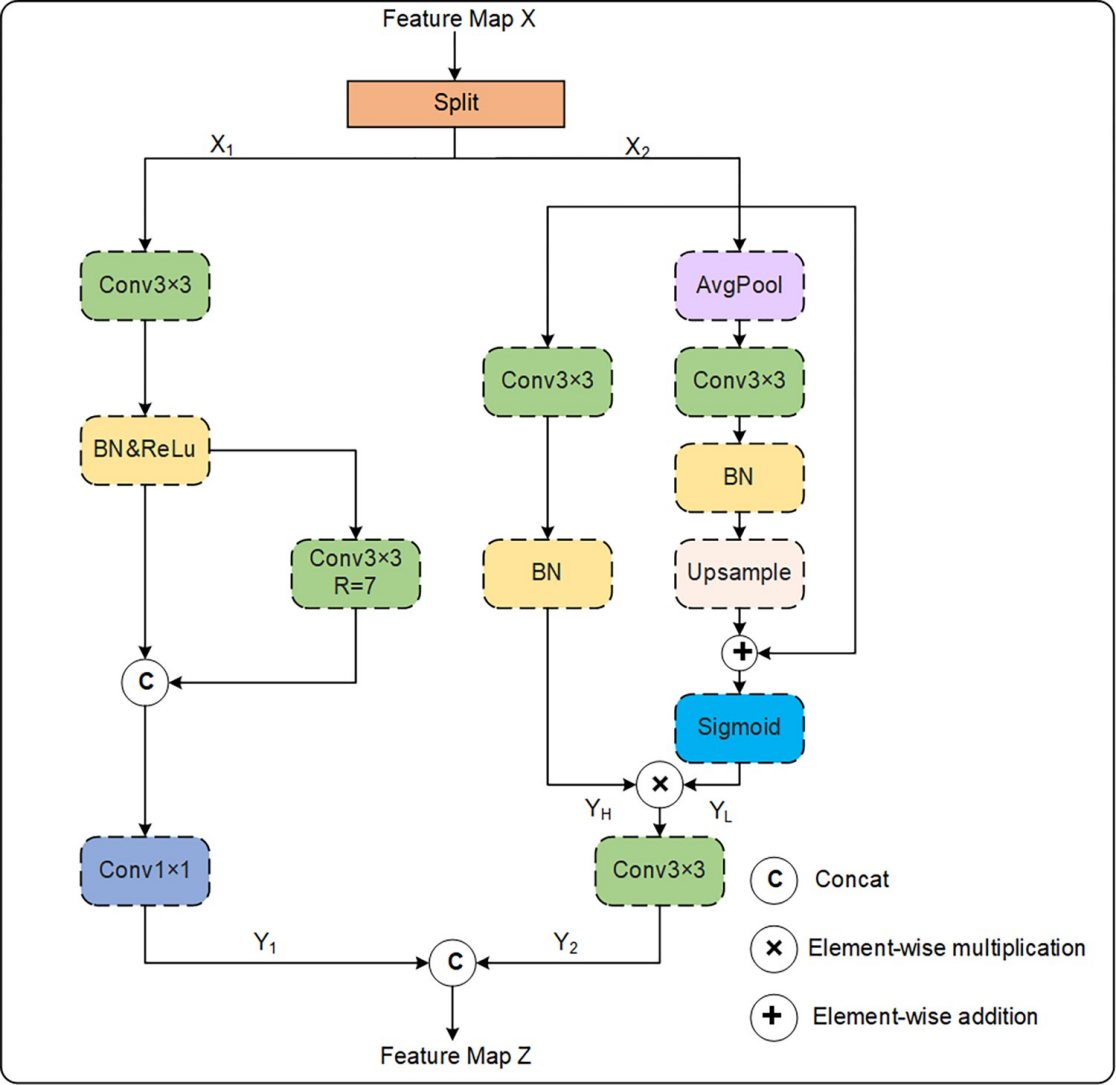
The SCFCConv structure, utilized by the proposed AFCF-Net network.

In the traditional U-Net architecture, 3×3 convolutions are used as a backbone to capture local features of the images [10]. However, in the task of skin lesion segmentation, different types of skin lesions have different sizes and shapes. Excessive capture of detailed features can lead to the network’s difficulty in accurately understanding the structural features of skin lesions. Self-calibrating convolution [28] introduces a self-calibration mechanism, allowing a network to automatically learn and adjust feature weights during training to better match lesion areas, thus improving the network’s adaptability to different types of lesion features. Inspired by self-calibrating convolution [28], we developed a novel type of convolution, named SCFCConv, to replace the second 3×3 convolution in each layer of the original U-Net. This improvement helps the network to more comprehensively extract semantic information of different types from the images and establish the dependency relationship between spatial and channel features. The structure of SCFCConv is illustrated in Fig 2.

To obtain different types of feature information from the input feature map, the input feature map *X*∈ℝ^C×H×W^ is evenly divided into two parts {*X*_1_, *X*_2_} along the channel dimension. These are separately fed into two different branches. In the first branch, a 3×3 convolution and a dilated convolution with a dilation rate of 7 are used to comprehensively capture both local features of lesions and more extensive spatial features. Subsequently, the features obtained from these two different receptive fields are concatenated, and a 1×1 convolution is used to adjust feature weights, resulting in the feature map *Y*_1_∈ℝ^C/2×H×W^ with rich local and global features. The above process is represented as follows:

$$X_{1},X_{2}=S(X)$$ (4)

$$Y_{1}=C_{1}(C_{3}^{7}(C_{3}(X_{1}))\|C_{3}(X_{1}))$$ (5)

where *S* represents partitioning the feature maps in the channel dimension, $C_{i}^{j}$ denotes a convolutional operation with a kernel size *i* and a dilation rate *j*, and ‖ represents concatenating the feature maps in the channel dimension.

The second branch controls the range of information acquisition by introducing contextual information around each position, enabling the network to better capture the dependency between features and reduce the acquisition of interference and noise. Features from the feature map *X*_2_ are extracted in two different resolution spaces. Firstly, a low-resolution space is generated by average pooling with a kernel size of 2, and then low-resolution features are smoothed and re-integrated using a 3×3 convolution. Subsequently, the feature maps are restored to their original size through upsampling operations. Additionally, a shortcut residual connection is used to preserve the original information in the feature map. Finally, the feature map *Y*_*L*_ is obtained through a sigmoid operation, which is used to guide the acquisition of key features in the high-resolution space. In this space, features are further extracted through convolutional operations and multiplied with *Y*_*L*_ to highlight and retain important features. Subsequently, the dependency between features is captured through a 3×3 convolution to obtain the feature map *Y*_2_. Finally, *Y*_1_ and *Y*_2_ are combined to obtain the output feature map *Z*∈ℝ^C×H×W^ of SCFCConv. The above process can be represented as follows:

$$Y_{2}=C_{3}(C_{3}(X_{2})*\sigma (U(C_{3}(A_{2}(X_{2})))+X_{2}))$$ (6)

$$Z=Y_{1}\|Y_{2}$$ (7)

where *σ* denotes the sigmoid function, * denotes an element-wise multiplication, *U* denotes an upsampling operation, and *A*_2_ denotes an average pooling operation with a kernel size of 2.

### 3.3 Feature symmetric fusion convolution

**Fig 3:**
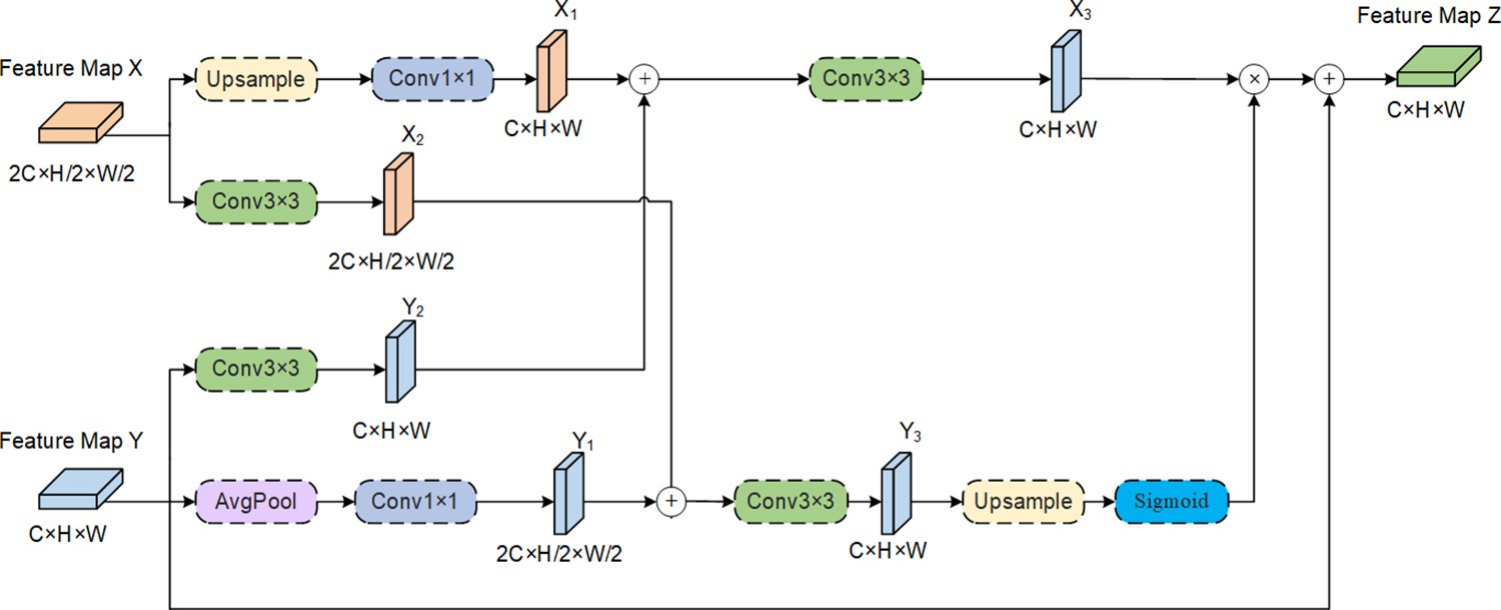
The FSFConv structure, utilized by the proposed AFCF-Net network.

High-frequency features reflect local changes in images, including detailed information such as boundaries and textures of lesions, while low-frequency features reflect global changes in images, mainly containing information about the overall structure of lesions [29]. Although AFCF-Net adopts SCFCConv as a backbone structure and successfully captures the structural features of skin lesions, there are still some deficiencies in obtaining boundary features and texture details in lesion areas. To address this issue, a novel type of convolution, named FSFConv, is proposed here, as shown in Fig 3. FSFConv aims to emphasize the extraction of high-frequency features while reducing feature redundancy, thereby enhancing the network’s perception of target boundaries and texture details, and more accurately preserving and restoring the target boundary details.

In SCFConv, a symmetrical feature extraction strategy is first employed to obtain diverse feature representations from the high-level feature *X*∈ℝ^2C×H/2×W/2^ and low-level feature *Y*∈ℝ^C×H×W^, resulting in feature maps {*X*_1_, *Y*_2_} and feature maps {*X*_2_, *Y*_1_}, respectively. Subsequently, the feature groups {*X*_1_, *Y*_2_} and {*X*_2_, *Y*_1_} are separately cross-fused to form feature maps *X*_3_ and *Y*_3_ containing richer multi-level information. Since the early feature maps of the network have higher resolution, retaining more detailed information in images, upsampling is used to adjust the size of the feature map *Y*_3_. Then, the sigmoid function is used to distinguish features of different frequencies. The results is multiplied with the high-frequency feature map *X*_3_ to enhance the high-frequency features in the early feature maps of the network. The final result is added to the feature map Y to highlight the lesion boundary features and texture details in the original feature map. The above process can be represented as follows:

$$X_{1}=C_{1}(U(X))$$ (8)

$$X_{2}=C_{3}(X)$$ (9)

$$Y_{1}=C_{1}(A_{2}(Y))$$ (10)

$$Y_{2}=C_{3}(Y)$$ (11)

$$Z=C_{3}(X_{1}+Y_{2})*\sigma (U(C_{3}(X_{2}+Y_{1})))+Y$$ (12)

### 3.4 Feature attention recombination

**Fig 4:**
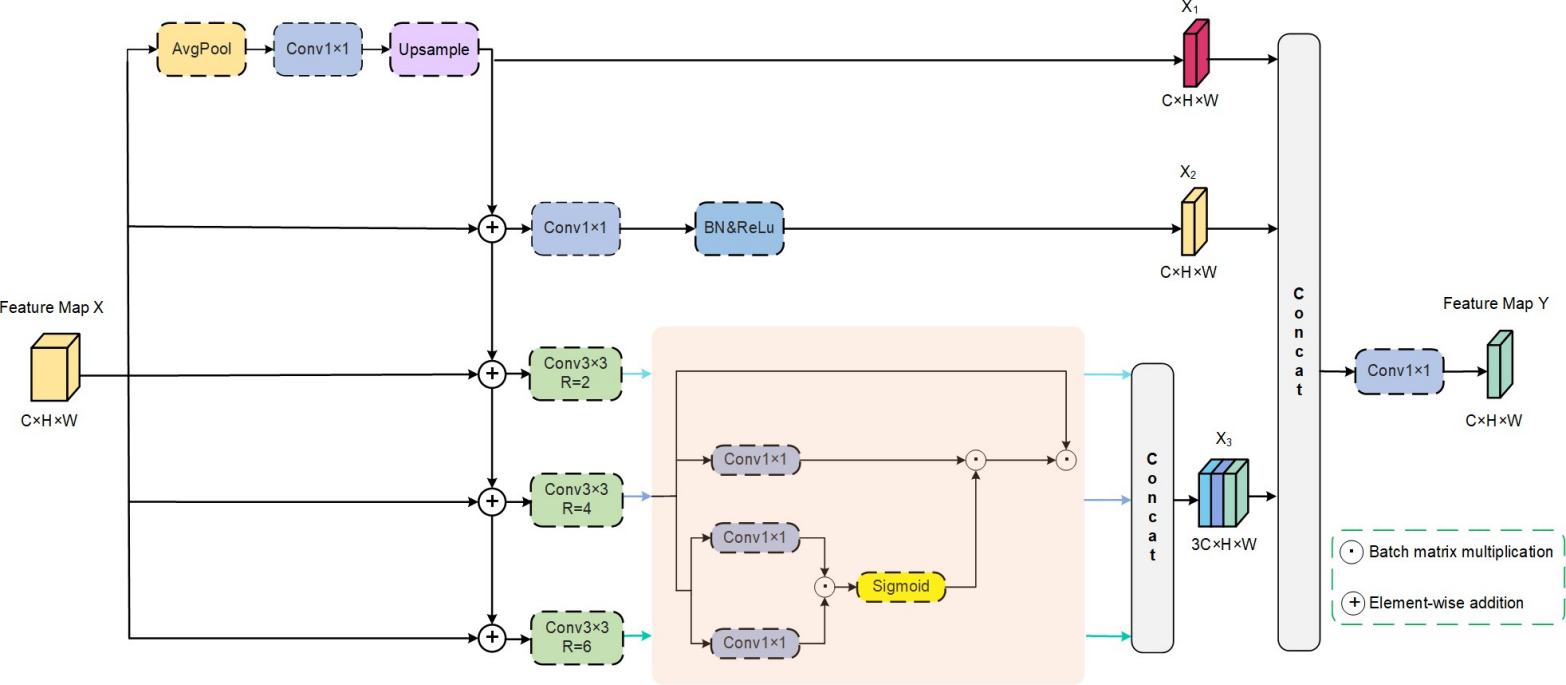
The FARM structure, utilized by the proposed AFCF-Net network.

In order to better handle visually significant regions of different scales in images and enhance the network’s understanding of lesions at various scales, this paper proposes a newly designed FARM, shown in Fig 4, which enhances feature fusion, based on the ASPP module [30]. By introducing dense multi-scale feature acquisition and visual attention mechanism, FARM can adaptively learn multi-scale features in input images, and make the network pay more attention to relatively important feature channels in multi-scale features, suppressing unimportant feature information, thereby enhancing the expression capability of multi-scale features. Additionally, by introducing feature recombination operations, different scale feature maps are reassembled to better facilitate interaction between features and the transmission of information.

Specifically, FARM initially preserves key features within the input feature map via a combination of pooling, convolution, and upsampling, and then fuses them with the original feature map. Subsequently, the parallel combination of a 1×1 convolution and convolutions with different expansion rates is employed to effectively extract multi-scale features across different receptive fields without introducing additional computational complexity. However, if significant regions in skin lesion images are not concentrated, directly aggregating features at different scales may weaken the representation ability of features in important areas [31]. Therefore, an attention module is introduced into each dilated convolution branch to calculate attention weights, based on the spatial feature relationships between arbitrary points in the feature map, enabling the network to focus on visually important regions in images, thereby enhancing the representation capabilities of features. It is worth noting that, in order to preserve the inherent characteristics of the other branch inputs, the attention modules are introduced only after each dilated convolution. Finally, the attention-enhanced feature maps are merged with the results of the other two branches. The output feature map of FARM is then obtained by mapping the feature information through a 1×1 convolution. The pseudo-code for the above process is provided in Algorithm 1.

Algorithm 1: The PyTorch-style pseudo-code of FARM

#Input: X, the feature map with shape [B, C, H, W]

#Output: Y, the feature map with shape [B, C, H, W]

#Operator: U, Bilinear interpolation

  AP, Average pooling operation

  Conv1: 1×1 convolution + Batch Normalization + ReLU

  Conv3: 3×3 convolution + Batch Normalization + ReLU, the second parameter represents the dilation rate, defaulting to 1 if not specified.

 1: X1 = U (Conv1 (AP (X)))

 2: X2 = Conv1 (X + X1))

 3: A = Conv3 (X + X1, 2)

 4: A = torch.bmm (torch.bmm (torch.sigmoid (torch.bmm (Conv1 (A), Conv1 (A))), Conv1 (A)), A)

 5: B = Conv3 (X + X1, 4)

 6: B = torch.bmm (torch.bmm (torch.sigmoid (torch.bmm (Conv1 (B), Conv1 (B))), Conv1 (B)), B)

 7: C = Conv3 (X + X1, 6)

 8: C = torch.bmm (torch.bmm (torch.sigmoid (torch.bmm (Conv1(C), Conv1 (C))), Conv1 (C)), C)

 9: X3 = torch.cat ([A, B, C], dim = 1)

 10: Y = Conv1 (torch.cat ([X1, X2, X3], dim = 1))

### 3.5 Multi-level feature aggregation

**Fig 5:**
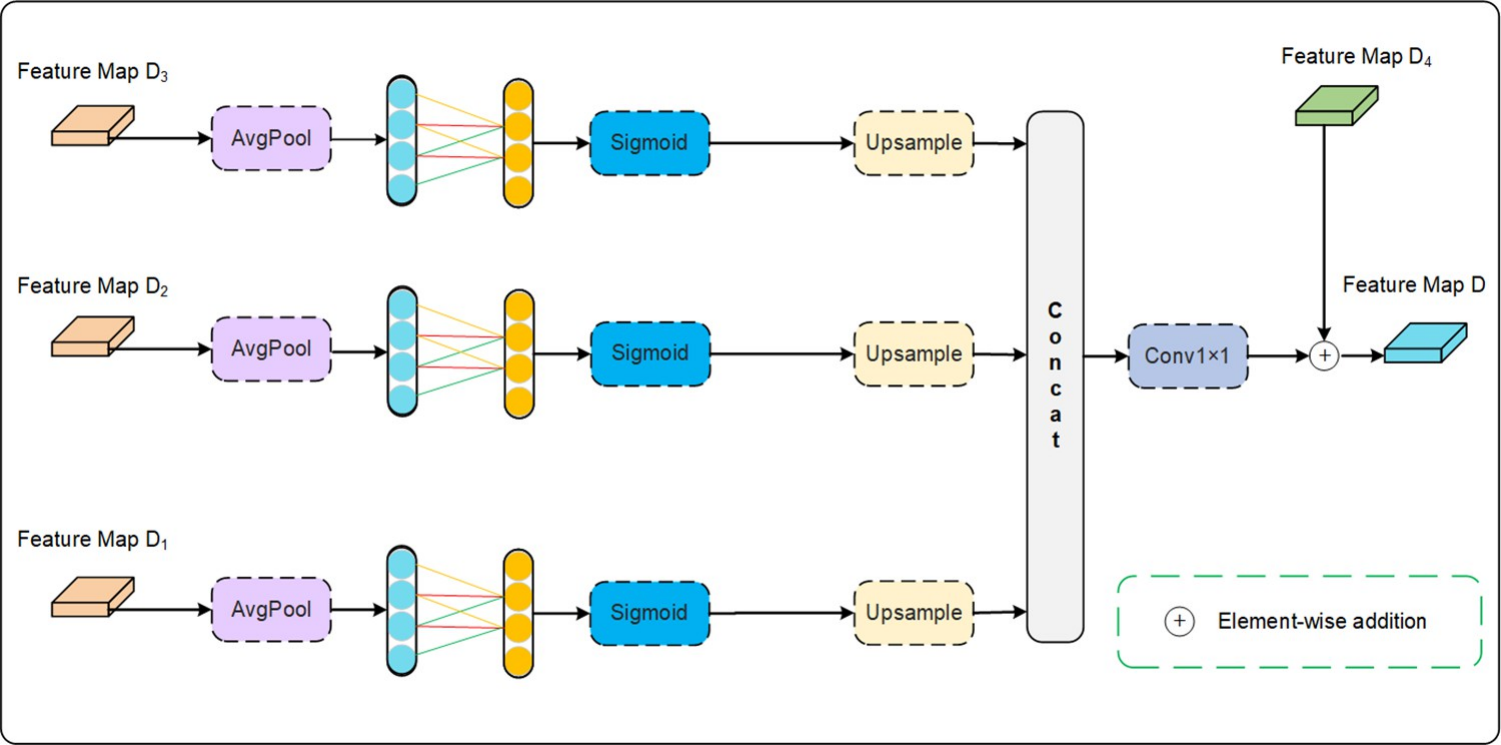
The structure of the multi-level feature aggregation branch, utilized by the proposed AFCF-Net network.

A single decoder limits the network’s ability to represent features at different scales, leading to network’s inability to accurately handle skin lesion images with multi-scale structures or complex backgrounds [32]. To introduce multi-level features, and enhance the flexibility and generality of the network, we developed a multi-level feature aggregation branch as an additional decoder for the proposed AFCF-Net network. Fig 5 illustrates the specific implementation details of this newly-designed branch.

First, considering that ECA attention helps a network better understand the correlation between features and extract important global information with lower computational cost [23], the output features {*D*_1_, *D*_2_, *D*_3_} of the first three layers of the original U-Net decoder are sent to ECA for processing, in order to enhance the representation of global features. It is worth noting that in the designed multi-level feature aggregation branch, only the visual attention part of the ECA attention is used, removing the residual multiplication with the original features. This is done to improve the independence of features, enabling the network to focus more on important information in the input features and reduce the learning of redundant features. Next, the feature maps are reconstructed and restored through upsampling. Since each decoder layer can capture information from different levels and scales of the images, features from different decoder layers are merged through a concatenation operation, and the image features are redefined through 1×1 convolution. Finally, the output features of the original decoder *D*_4_ are fused to complete the supplementation of the original decoder features.

Integrating the multi-level feature aggregation branch into the network can alleviate the problem of segmentation performance degradation caused by information loss during network propagation. Especially during the multi-level feature fusion process, preserving more detailed information helps improve the segmentation performance of the network and enhances its generalization ability.

## 4 Experiments and results

### 4.1 Datasets and data preprocessing

To validate the segmentation performance of AFCF-Net, experiments were conducted on three public datasets (ISIC2017 [33], ISIC2018 [34], PH2 [35]) and a private dataset. ISIC2017 is a publicly available dataset, used for skin disease segmentation and classification, covering various types of skin diseases such as melanoma, squamous cell carcinoma, etc., demonstrating certain diversity and representativeness. To ensure fairness and rigor of the experiments, from this dataset we selected 2150 dermoscopic images commonly used for experimentation in skin lesion segmentation tasks [17, 36, 37]. The ISIC2018 dataset, provided by the International Skin Imaging Collaboration (ISIC) 2018 challenge, is widely used for skin lesion classification and segmentation tasks. It contains 2594 skin lesion images. The PH2 dataset contains a total of 200 images, which were used as an additional test set to ISIC2017. The private dataset was provided by Peking Union Medical College Hospital and consists of 1100 images of skin diseases. The labels corresponding to these images were manually annotated under the guidance of professional physicians. The feature performance of the private dataset is not as significant as that of the public datasets, which means that a network needs to learn more subtle features in order to accurately segment the lesion areas.

In the experiments, the ISIC2017 and ISIC2018 public datasets, along with the private dataset, were divided into training, test, and validation sets using a ratio of 7:2:1. Before network training, preprocessing such as random rotation and adding noise were applied to the training images to prevent network overfitting.

### 4.2 Experimental environment

The experiments were conducted on a computer equipped with a single Xeon(R) Platinum 8255C CPU and an RTX 3080 GPU, using the PyTorch framework. During the training process, the combined BCE-Dice loss was used for network training. The epoch number was set to 200, the batch size was set to 8, the initial learning rate was set to 1e-4, and the learning rate decay factor was 0.5.

### 4.3 Evaluation metrics

To evaluate the segmentation performance of AFCF-Net in comparison with existing networks, four performance metrics were employed, whereby the Intersection over Union (IoU) and the Dice Similarity Coefficient (DSC) served as the primary evaluation metrics, while accuracy (Acc) and sensitivity (Sen) were used to assist in judging the network segmentation performance. The values of these metrics were calculated as follows:

$$IoU=\frac{TP}{FP+TP+FN}$$ (13)

$$DSC=\frac{2TP}{2TP+FP+FN}$$ (14)

$$Acc=\frac{TP+TN}{TP+TN+FP+FN}$$ (15)

$$Sen=\frac{TP}{TP+FN}$$ (16)

where *TP* (true positives) denotes the number of pixels accurately identified by a network as part of a skin lesion area, *FP* (false positives) denotes the number of pixels incorrectly identified by a network as part of a skin lesion area, *FN* (false negatives) denotes the number of pixels incorrectly identified by a network as not being part of a skin lesion area, and *TN* (true negatives) denotes the number of pixels correctly identified by a network as not being part of a skin lesion area.

### 4.4 Results and analysis

#### 4.4.1 Performance comparison on ISIC2017 dataset

In the first group of experiments, we compared AFCF-Net with several classical networks (U-Net [10], UNet++ [11], Attention U-Net [38]), lightweight networks (MALUNet [16], EGE-UNet [36]), and complex networks (TransUNet [39], DCSAU-Net [40]) on the ISIC2017 dataset. The experimental results are shown in Table 1 (the best result for each metric is highlighted in **bold**). It is evident from this table that AFCF-Net achieves state-of-the-art performance, being ranked first by all evaluation metrics among all compared networks. Specifically, the proposed network achieved 83.55% and 90.24% on the two major evaluation metrics in the field of image segmentation (i.e., IoU and DSC), by outperforming the second-ranked DCSAU-Net (and Attention U-Net in the case of DSC) by 1.05 and 1.10 percentage points, respectively. Additionally, compared to some larger networks, AFCF-Net exhibits significantly reduced number of parameters and computational complexity, requiring only 0.39M parameters and achieving 0.41G Floating Point Operations Per Second (FLOPS), approaching the level of lightweight networks. For instance, compared to TransUNet, the number of AFCF-Net parameters is less by approximately 260 times. Overall, while the proposed network does not possess fewer parameters or lower computational complexity, compared to lightweight networks such as MALUNet and EGE-UNet, it significantly surpasses them in segmentation performance. Moreover, AFCF-Net even outperforms mainstream large networks.

**Table 1:** Skin lesion segmentation performance results of networks, achieved on the ISIC 2017 dataset (based on experiments).

Networks	Params (M)	FLOPS (G)	IoU (%)	DSC (%)	Acc (%)	Sen (%)
U-Net	7.23	12.14	81.08	88.37	94.38	90.80
UNet++	9.16	34.90	81.91	88.98	94.69	90.15
Attention U-Net	34.87	66.63	81.75	88.87	94.39	90.17
TransUNet	100.99	34.54	82.25	89.14	94.85	90.04
MALUNet	0.17	0.08	79.69	87.45	93.98	89.05
EGE-UNet	**0.04**	**0.07**	79.63	87.02	93.68	88.15
DCSAU-Net	2.59	6.91	82.50	89.14	94.36	90.90
AFCF-Net (*proposed*)	0.39	0.41	**83.55**	**90.24**	**95.06**	**91.03**

**Fig 6:**
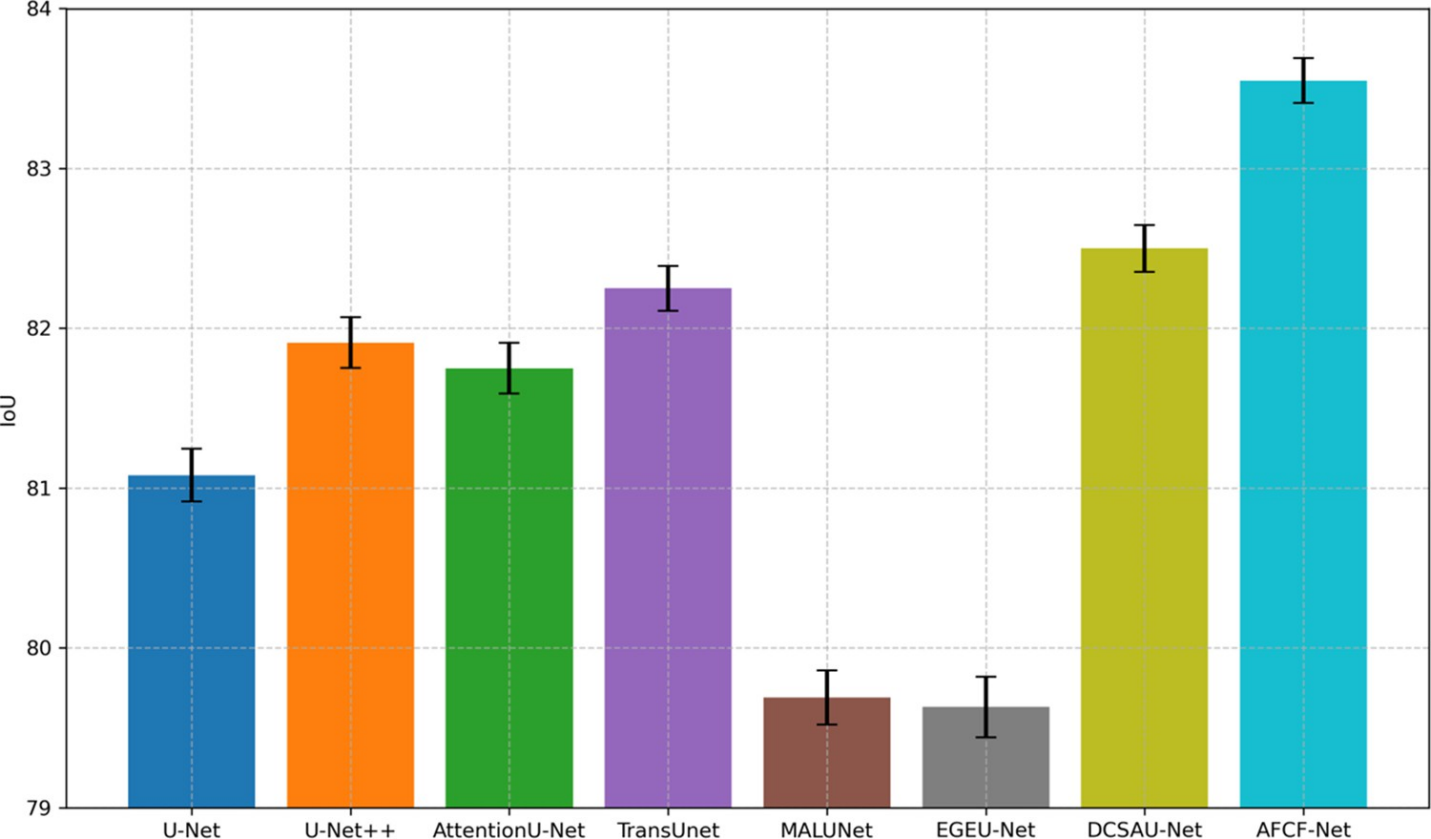
Average IoU and IoU standard deviation of networks, compared on the ISIC2017 dataset.

Fig 6 shows the average IoU value (top of the corresponding bar) and IoU standard deviation (short vertical line on the bar) of each network, compared on the ISIC2017 dataset, based on five conducted experiments. It is evident from this figure that the average IoU of the proposed AFCF-Net network is significantly higher than that of other networks, indicating its superior overall performance in this task, allowing for more accurate identification and segmentation of skin lesion areas.

**Fig 7:**
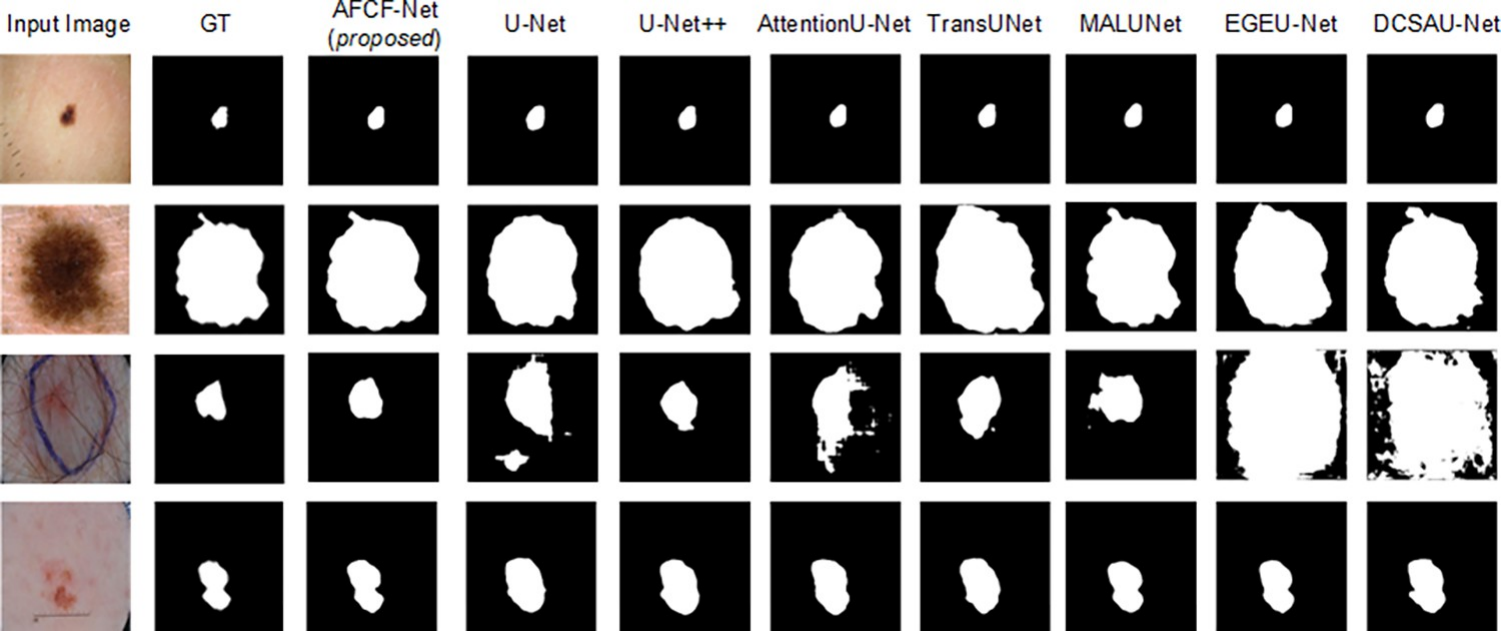
Visualization of skin lesion segmentation results of compared networks, achieved on the ISIC2017 dataset.

Fig 7 visualizes the skin lesion segmentation results of the networks, compared on the ISIC2017 dataset. From this figure, it becomes more intuitive to observe that when segmenting images, containing interference from hair or other distractors (as depicted in the third row of Fig 7), other networks tend to misclassify the distractors as lesion areas. In contrast, the proposed AFCF-Net network excludes the influence of these interferences and accurately segments the true lesion areas. Additionally, when confronted with images where the lesion areas are small, large, or not very distinct (corresponding to the first, second, and fourth rows of Fig 7), the segmentation results of AFCF-Net are closer to the ground-truth labels compared to other networks.

Additionally, we also compared AFCF-Net with some of the state-of-the-art non-open-source networks. The experimental results of these networks are taken from the respective literature sources, indicated in Table 2 (missing data in a source are represented by "-"; the best result for each metric is highlighted in **bold**). As can be seen from this table, AFCF-Net is still in a leading position according to both major performance metrics, i.e., IoU and DSC, where DAU-Net [32] performs second, lagging back by 0.19 and 1.02 percentage points, respectively, while its parameter count is significantly higher than that of the proposed network. Compared to the Transformer-based FITrans network [41], AFCF-Net outperforms it by 1.85, 1.44, 0.36, and 4.63 percentage points, respectively, based on the four performance metrics. Compared to the larger ADTF network [42], the proposed network scores 2.14 percentage points higher on IoU and 1.60 percentage points higher on DSC. This indicates that AFCF-Net has extremely strong competitiveness among various types of mainstream networks in use today.

**Table 2:** Skin lesion segmentation performance results of networks, achieved on the ISIC 2017 dataset (based on literature sources).

Networks	Params (M)	FLOPS (G)	IoU (%)	DSC (%)	Acc (%)	Sen (%)
IESBU-Net [37]	**0.24**	**0.09**	79.54	88.12	96.38	**92.62**
ADTF [42]	8.60	-	81.41	88.64	**96.60**	-
MSMA-Net [43]	-	-	78.94	86.83	94.17	-
I-UNext [44]	1.48	2.78	71.10	82.46	92.86	76.01
FITrans [41]	-	-	81.70	88.80	94.70	86.40
DAU-Net [32]	3.50	-	83.36	89.22	-	-
Lama et al. [45]	-	-	80.70	88.00	94.80	-
TC-Net [46]	-	-	74.55	85.20	93.68	81.45
HDS-Net [47]	-	-	77.50	85.70	-	85.20
AFCF-Net (*proposed*)	0.39	0.41	**83.55**	**90.24**	95.06	91.03

#### 4.4.2 Performance comparison on PH2 dataset

The second group of experiments transferred the training results of each network participating in the first group of experiments, conducted on the ISIC2017 dataset (c.f., Table 1), to the PH2 dataset for comparison of the network generalization ability. The obtained results are shown in Table 3.

**Table 3:** Skin lesion segmentation performance results of networks, achieved on the PH2 dataset (based on experiments).

Networks	Params (M)	FLOPS (G)	IoU (%)	DSC (%)	Acc (%)	Sen (%)
U-Net	7.23	12.14	80.65	88.78	92.76	94.83
UNet++	9.16	34.90	82.28	89.83	93.26	95.09
Attention U-Net	34.87	66.63	82.45	89.93	93.39	94.81
TransUNet	100.99	34.54	82.93	89.82	93.58	94.45
MALUNet	0.17	0.08	82.19	89.72	93.21	94.83
EGE-UNet	**0.04**	**0.07**	82.54	89.66	92.86	93.08
DCSAU-Net	2.59	6.91	83.86	90.55	93.93	**96.19**
AFCF-Net (*proposed*)	0.39	0.41	**84.81**	**91.26**	**94.00**	95.47

In this set of experiments, AFCF-Net achieved IoU, DSC, accuracy, and sensitivity values of 84.81%, 91.26%, and 94.00%, respectively, which were 0.95, 0.71, and 0.07 percentage points higher than those of the second-ranked DCSAU-Net. However, the AFCF-Net’s sensitivity (95.47%) did not reach the highest ranking, lagging behind the first-placed sensitivity of DCSAU-Net by 0.72 percentage points.

**Fig 8:**
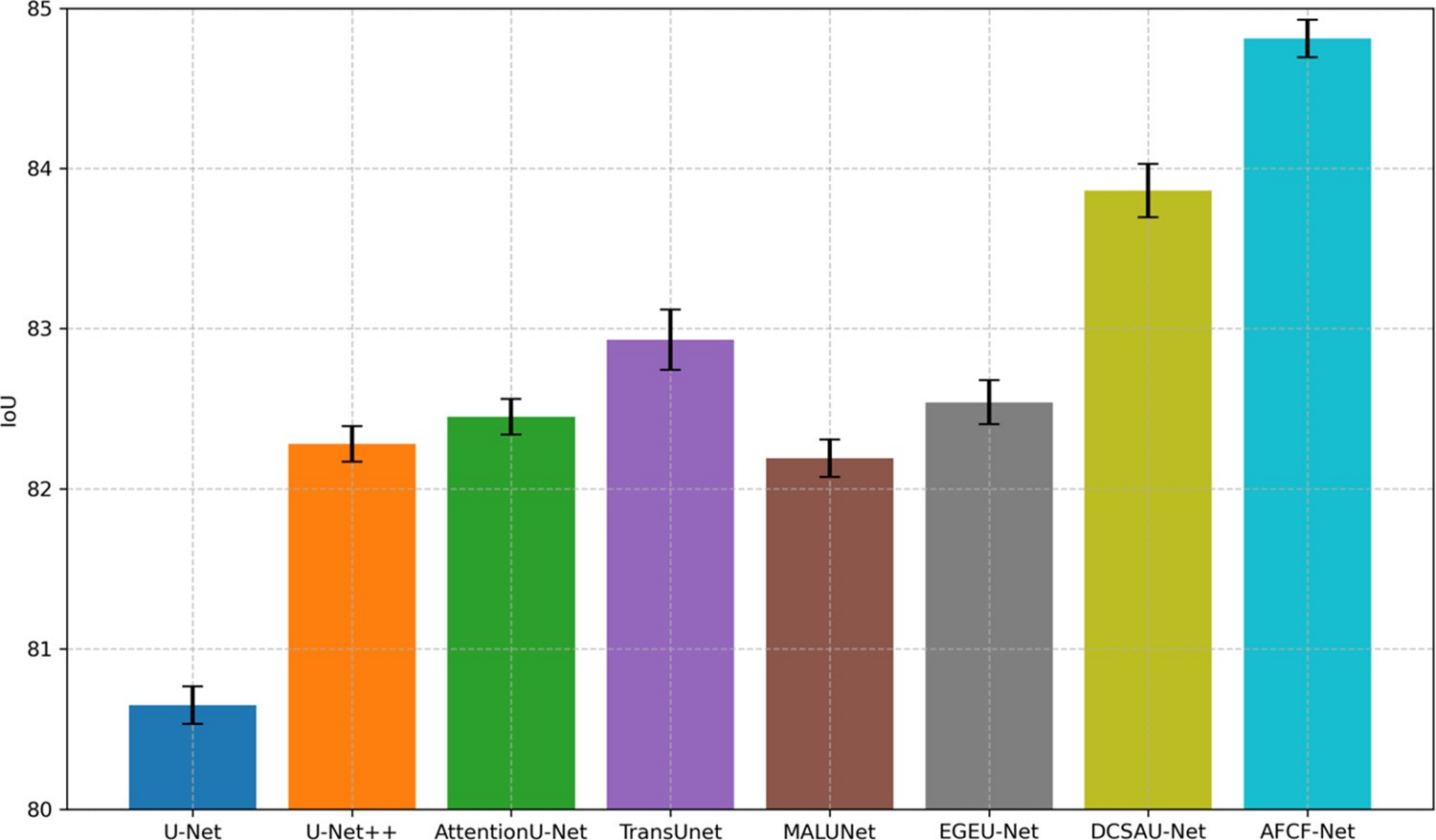
Average IoU and IoU standard deviation of networks, compared on the PH2 dataset.

Fig 8 shows a visual comparison of the average IoU and IoU standard deviation of the networks, compared in this cross-dataset testing. As can be seen from this figure, the segmentation performance of AFCF-Net still maintains first position, indicating that the proposed network has strong generalization ability and can adapt to data with different characteristics, rather than being limited to specific datasets. In addition, compared with testing on a single dataset, testing on cross-datasets causes the results of some networks to fluctuate significantly. For example, the standard deviations of TransUNet and DCSAU-Net are significantly higher than those of other networks, indicating that these networks are unstable when processing image features never been seen before, and thus need more data to improve their consistency. Overall, the stable performance of AFCF-Net on different datasets demonstrates its superior generalization ability, making it more reliable and adaptable in practical applications.

**Fig 9:**
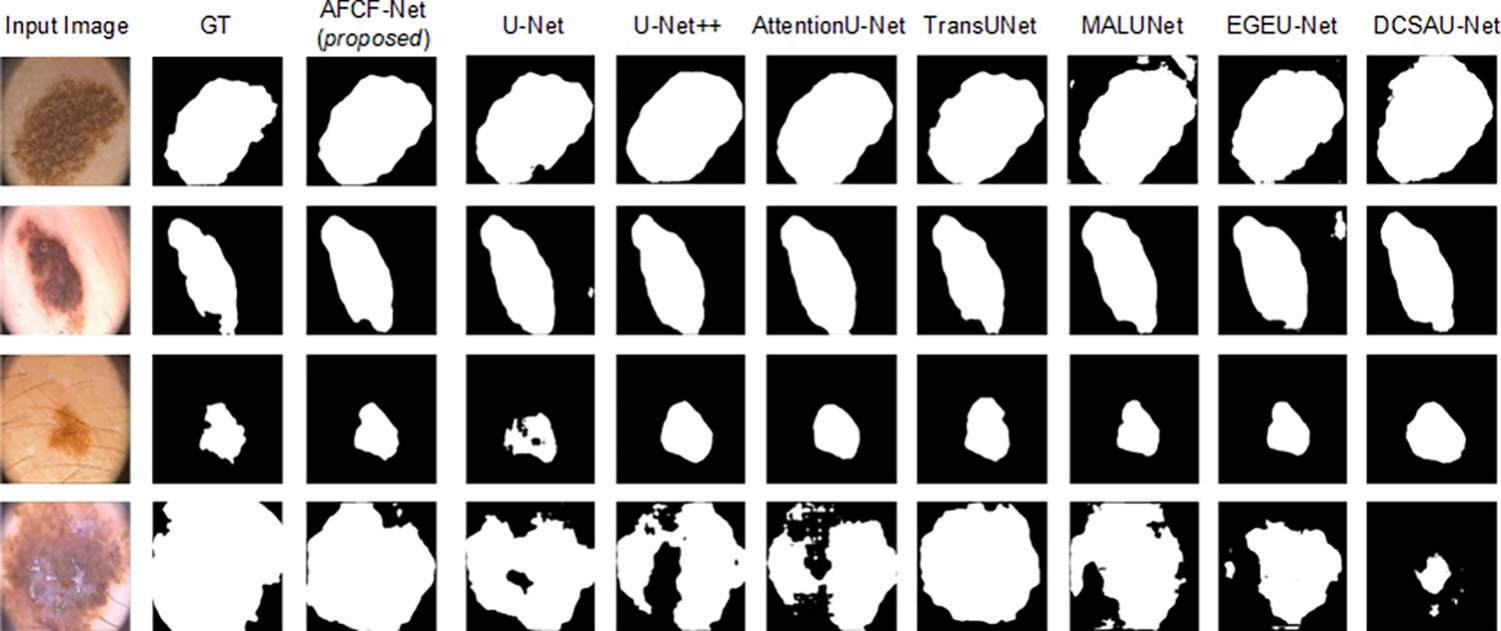
Visualization of skin lesion segmentation results of compared networks, achieved on the PH2 dataset.

Fig 9 visualizes the skin lesion segmentation results of the networks, compared on the PH2 dataset. From this figure, it can be observed that in the case of multiple lesion overlaps, shown in the fourth row, DCSAU-Net excessively focuses on regions with prominently featured lesion characteristics, thereby overlooking some relatively less prominent lesion features. However, AFCF-Net can comprehensively consider various lesion information and accurately segment complete lesion areas. Overall, compared to other networks, the proposed network can respond more flexibly and effectively to diverse types of data and features. This indicates that AFCF-Net has an advantage in handling untrained lesion scenarios, as it can more comprehensively capture various types of lesion features, thereby improving the quality and accuracy of segmentation results.

#### 4.4.3 Performance comparison on ISIC2018 dataset

**Fig 10:**
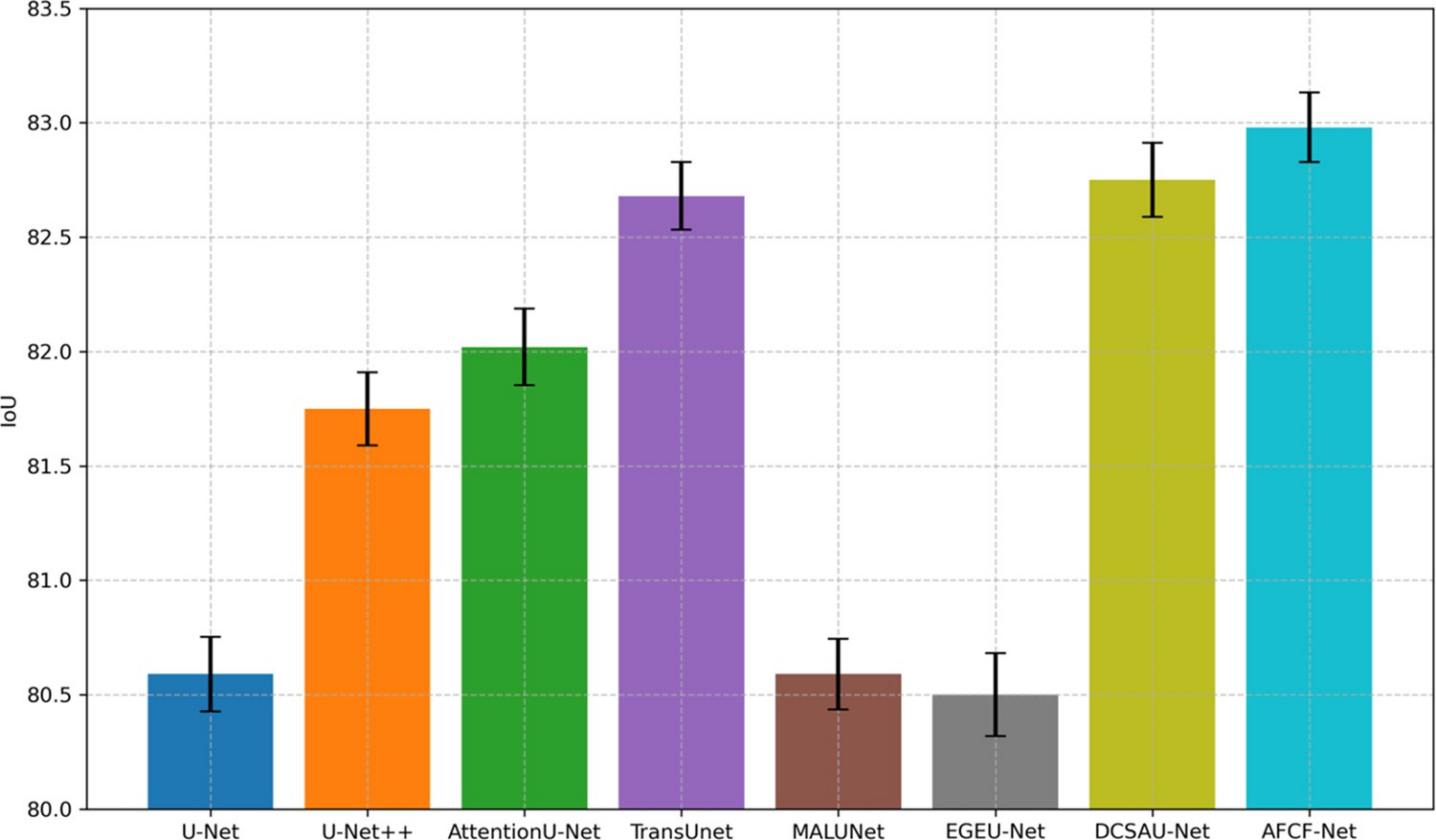
Average IoU and IoU standard deviation of networks, compared on the ISIC2018 dataset.

To evaluate the performance of AFCF-Net across multiple datasets, in the third set of experiments, we compared AFCF-Net with the same set of networks, participated in the first two sets of experiments, on a third dataset, namely the ISIC2018 public dataset. The experimental results and mean error statistics are presented in Table 4 and Fig 10, respectively. From these, one can see that there are significant differences in segmentation performance between lightweight and large networks on this dataset. This is because large networks, like TransUNet, increase the number of channels to enhance the depth of feature maps, allowing them to extract and retain more feature information at each layer. Multiple channels capture richer image details and texture information, thereby improving the network segmentation performance. In contrast, lightweight networks reduce the number of channels to lower their computational complexity, which, however, leads to a loss of information. Compared to other networks, AFCF-Net achieves higher segmentation performance with lower network complexity, achieving the best performance on this dataset, according to three (out of four) evaluation metrics used. The AFCF-Net’s IoU, DSC, and accuracy reached 82.92%, 89.72%, and 95.08%, respectively, ranking it first among the compared networks. Moreover, the smaller standard deviation (c.f., Fig 10) means that AFCF-Net provides relatively consistent segmentation results across different cases and images, resulting in higher diagnostic reliability.

**Table 4:** Skin lesion segmentation performance results of networks, achieved on the ISIC2018 dataset (based on experiments).

Networks	Params (M)	FLOPS (G)	IoU (%)	DSC (%)	Acc (%)	Sen (%)
U-Net	7.23	12.14	80.59	88.09	94.58	**93.53**
UNet++	9.16	34.90	81.75	88.84	94.67	90.83
Attention U-Net	34.87	66.63	82.02	88.90	94.81	90.29
TransUNet	100.99	34.54	82.68	89.62	94.94	90.62
MALUNet	0.17	0.08	80.59	88.26	94.33	90.57
EGE-UNet	**0.04**	**0.07**	80.50	87.72	94.17	89.15
DCSAU-Net	2.59	6.91	82.75	89.37	94.92	91.46
AFCF-Net (*proposed*)	0.39	0.41	**82.92**	**89.72**	**95.08**	89.64

**Fig 11:**
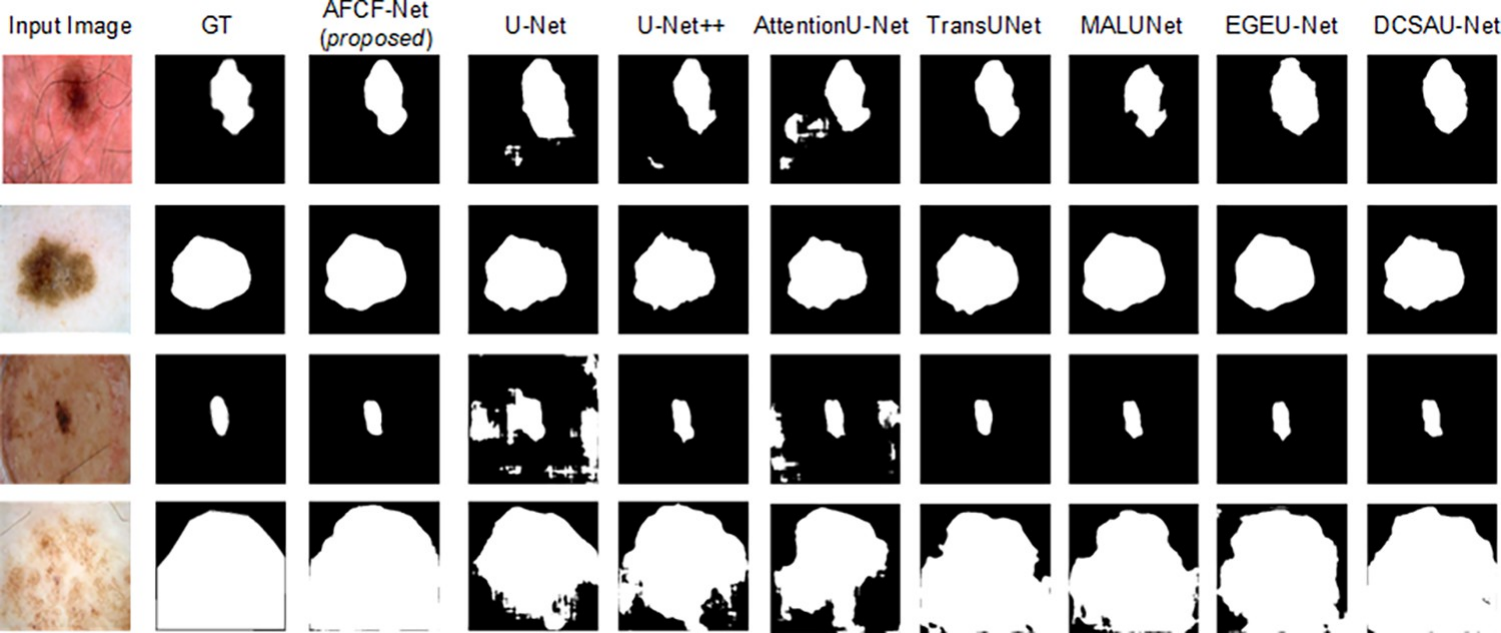
Visualization of skin lesion segmentation results of compared networks, achieved on the ISIC2018 dataset.

Fig 11 visualizes the skin lesion segmentation results of the networks, compared on the ISIC2018 dataset. From the first and second rows of this figure, it can be seen that AFCF-Net excels in accurately identifying and segmenting skin lesion boundaries, particularly well w.r.t. fine structures, where it significantly outperforms other networks. Additionally, AFCF-Net is more effective in handling lesion areas with complex backgrounds (third row of Fig 11). This is because AFCF-Net enhances the contrast between important features and background features by means of the newly elaborated FSFConv, reduces the interference of irrelevant features on segmentation results, and effectively fuses key features using the newly designed multi-level feature aggregation branch, leading to its better overall performance. Finally, the fourth row of Fig 11 displays the segmentation results of the compared networks when dealing with larger skin lesion areas. This comparison clearly shows that AFCF-Net segmentation results are more complete and accurate compared to those of the compared networks.

#### 4.4.4 Performance comparison on private dataset

In the four set of experiments, AFCF-Net was compared with the same group of mainstream networks on a privately owned dataset with greater segmentation difficulty. The experimental results are shown in Table 5 (the best result for each metric is highlighted in **bold**). From this table, it is obvious that on this dataset, AFCF-Net has once again achieved excellent segmentation performance, surpassing all mainstream networks, according to all evaluation metrics. Based on IoU, the second-placed network (DCSAU-Net) scored less by 0.28 percentage points, whereas according to DSC, accuracy, and sensitivity the second-placed network is TransUNet, lagging behind by 0.38, 0.03, and 1.15 percentage points, respectively.

**Table 5:** Generalization ability comparison results of networks, achieved on the private dataset.

Networks	Params (M)	FLOPS (G)	IoU (%)	DSC (%)	Acc (%)	Sen (%)
U-Net	7.23	12.14	60.56	73.09	91.03	78.27
UNet++	9.16	34.90	61.63	74.04	91.32	78.27
Attention U-Net	34.87	66.63	61.88	74.32	91.48	79.43
TransUNet	100.99	34.54	63.77	76.52	91.52	80.16
MALUNet	0.17	0.08	60.62	73.77	90.42	80.12
EGE-UNet	**0.04**	**0.07**	61.77	75.11	91.02	79.71
DCSAU-Net	2.59	6.91	63.79	76.15	91.38	79.29
AFCF-Net (*proposed*)	0.39	0.41	**64.07**	**76.90**	**91.55**	**81.31**

**Fig 12:**
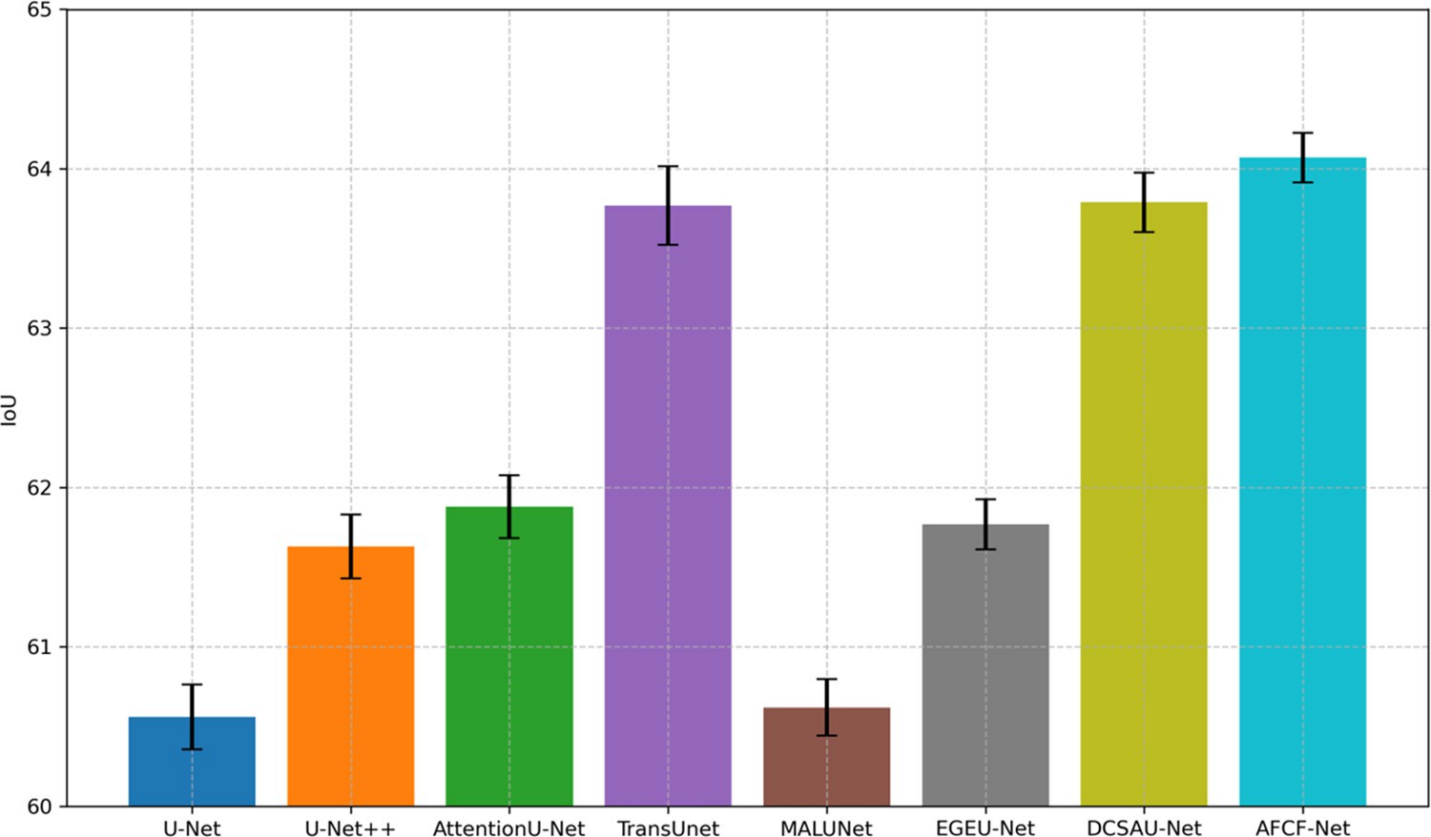
Average IoU and IoU standard deviation of networks, compared on the private dataset.

Additionally, as shown in Fig 12, some lightweight networks (e.g., MALUNet and EGE-UNet) exhibit high consistency on this dataset, with smaller standard deviations. In contrast, complex networks (e.g., TransUNet) exhibit larger standard deviations, indicating greater variability in the performance. This variability is due to the high complexity of Transformer-based networks, which require more data for adequate training compared to lightweight networks. However, the small size of this dataset makes it difficult for a network to fully learn the key features of skin lesions, leading to lower stability. In contrast, AFCF-Net achieves a balance between segmentation performance and network complexity, maintaining high stability and high performance even when dealing with small and complex datasets, demonstrating its adaptability and superiority in challenging practical scenarios.

**Fig 13:**
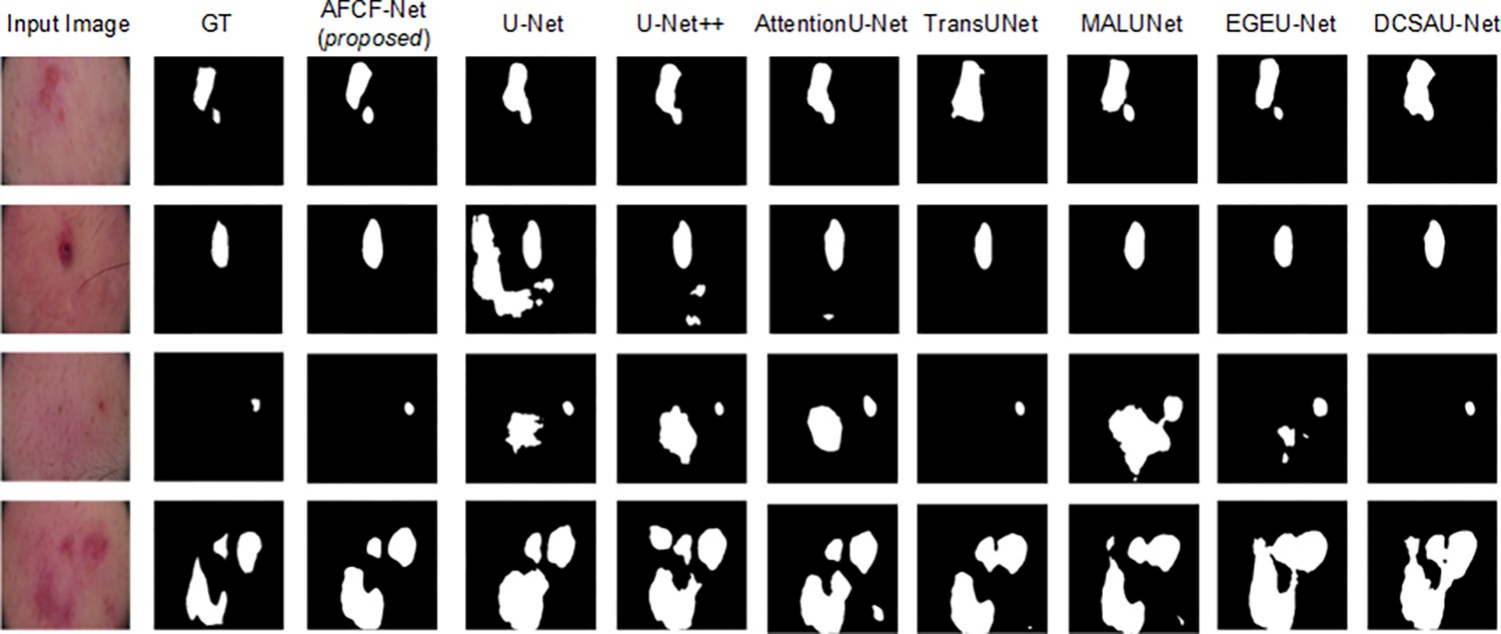
Visualization of skin lesion segmentation results of compared networks, achieved on the private dataset.

Through the visualization comparison of the skin lesion segmentation results of the compared network, shown in Fig 13, one can observe that other networks often mistakenly label some areas, which are not actually lesions, as lesion areas − a situation referred to as false positives (FP). In contrast, AFCF-Net performs better in this aspect, with fewer FP cases generated. This finding suggests that AFCF-Net achieved better performance and higher reliability in distinguishing between lesion and non-lesion areas. This advantage is mainly attributed to the two key newly elaborated components of AFCF-Net, namely FSFConv and FARM, which effectively introduce multi-scale information and enhance the contrast between high-frequency and low-frequency features. This allows the proposed network to more accurately learn the feature differences between lesion areas and background, resulting in even superior performance when dealing with relatively complex datasets.

#### 4.4.5 Ablation study

In order to verify whether the newly elaborated components and their combinations have a positive impact on the segmentation performance of the proposed network, we conducted ablation study experiments on the ISIC2017 dataset. In these experiments, we reduced the number of U-Net channels to {4, 8, 16, 32, 64}, and used this as a baseline network. The obtained experimental results are shown in Table 6.

**Table 6:** Results of ablation experiments.

Step	Network components	Inference time (s)	FLOPS (G)	IoU (%)	DSC (%)	Acc (%)	Sen (%)
0	Baseline	4.39	0.20	80.25	87.81	94.15	89.26
1	+ SCFCConv	5.41	0.21	81.38	88.64	94.29	90.35
2	+ FSFConv	5.25	0.34	81.58	88.64	94.24	90.49
3	+ FARM	5.00	0.24	81.29	88.29	94.16	**91.28**
4	+ SCFCConv + FSFConv	6.27	0.35	82.30	89.23	94.68	89.27
5	+ SCFCConv + FARM	6.07	0.25	82.07	89.03	94.63	88.97
6	+ FSFConv + FARM	6.08	0.38	82.24	89.10	94.34	89.17
7	+ SCFCconv + FSFConv + FARM	6.83	0.39	83.08	89.90	94.96	89.81
8	AFCF-Net (*proposed*)	7.25	0.41	**83.55**	**90.24**	**95.03**	91.03

First, the newly elaborated components (i.e., SCFCConv, FSFConv, and FARM) were individually added to the baseline to validate the effectiveness of each of them. From Table 6, it is evident that each component has brought about a certain degree of performance improvement to the network. This indicates that the three newly elaborated components can effectively enhance the network segmentation performance. Next, we added these components to the baseline in paired combinations. In this case, IoU, DSC, and accuracy have improved their values, compared to the single use of components, indicating that there is no redundancy issue between the components, and each of them can play a unique role in bringing positive effects to the network. Subsequently, after simultaneously integrating the three elaborated components into the baseline, the network performance was further improved, according to three (out of four) evaluation metrics. Finally, after adding the multi-level feature aggregation branch, along with to the three elaborated components (resulting in the proposed AFCF-Net network), three (out of the four) metrics reached their top values, with only sensitivity being lower than the sole use of FARM. Specifically, the two main metrics (IoU and DSC) reached 83.55% and 90.24%, respectively, and accuracy reached 95.03%. These experiments fully demonstrate the effectiveness of all newly elaborated components.

From the perspective of network’s inference speed, adding the newly elaborated components to the baseline does impact the inference speed to some extent, of course, but this impact is relatively minor compared to the significant improvements in the network segmentation performance and generalization ability. As shown in Table 6, the addition of FSFConv increased the network’s inference time from 4.39s to 5.25s; however, FLOPS did also increase from 0.20G to 0.34G. Although there is a slight increase in the computational overhead, FSFConv improves all metrics, indicating that FSFConv’s enhancement of segmentation performance significantly outweighs the increase in computational cost. Additionally, this component enhances the network’s ability to process boundary texture features. Another noteworthy aspect is the combination of SCFCConv and FARM, which demonstrates significant improvement in three metrics, including the two major one (IoU and DSC), compared to the standalone use of FARM. However, the inference time increase by only 1.07s, compared to FARM, indicating that this pair of components allows to significantly improve the network segmentation performance with only a minor increase in the computational overhead. Finally, after integrating all components into the baseline, resulting in the proposed AFCF-Net network, the inference time reached 7.25s but FLOPS reached 0.41G. Although the inference time and computational complexity have further increased, the overall performance of the proposed network demonstrate a significant improvement compared to the baseline. Overall, AFCF-Net demonstrates very good results in both segmentation performance and inference speed.

**Fig 14:**
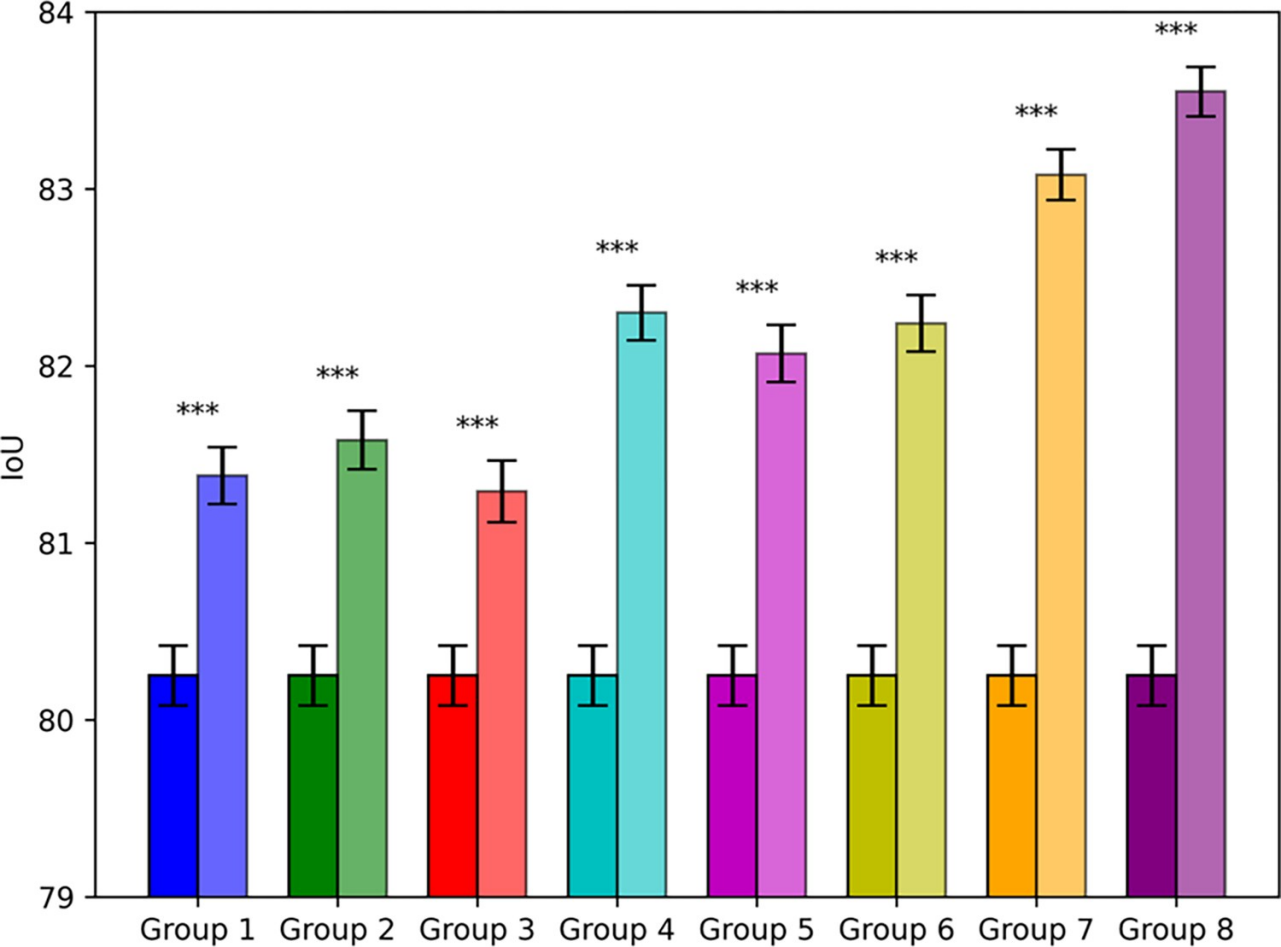
IoU significance comparison of newly elaborated components added to the baseline (group numbers correspond to step numbers in Table 6; the left column of each group represents the IoU of the baseline, whereas the right column represents the IoU of the resultant network after adding the corresponding component(s); the asterisks on each group represents the significance level).

Fig 14 depicts the IoU significance comparison of the newly elaborated components added to the baseline. It is evident from this figure that as components were gradually added to the baseline, the network’s standard deviation decreased, indicating that the newly elaborated components have brought greater stability to the baseline. Additionally, "***" in Fig 14 indicate the difference in the performance between the improved network and the baseline. The results show that the p-values for these differences are less than 0.001, indicating that the performance differences between the improved network and the baseline are highly significant, meaning that the effects of each newly elaborated component far exceed those caused by random fluctuations. This significant result provides strong support for the reliability of AFCF-Net in skin lesion segmentation tasks.

**Fig 15:**
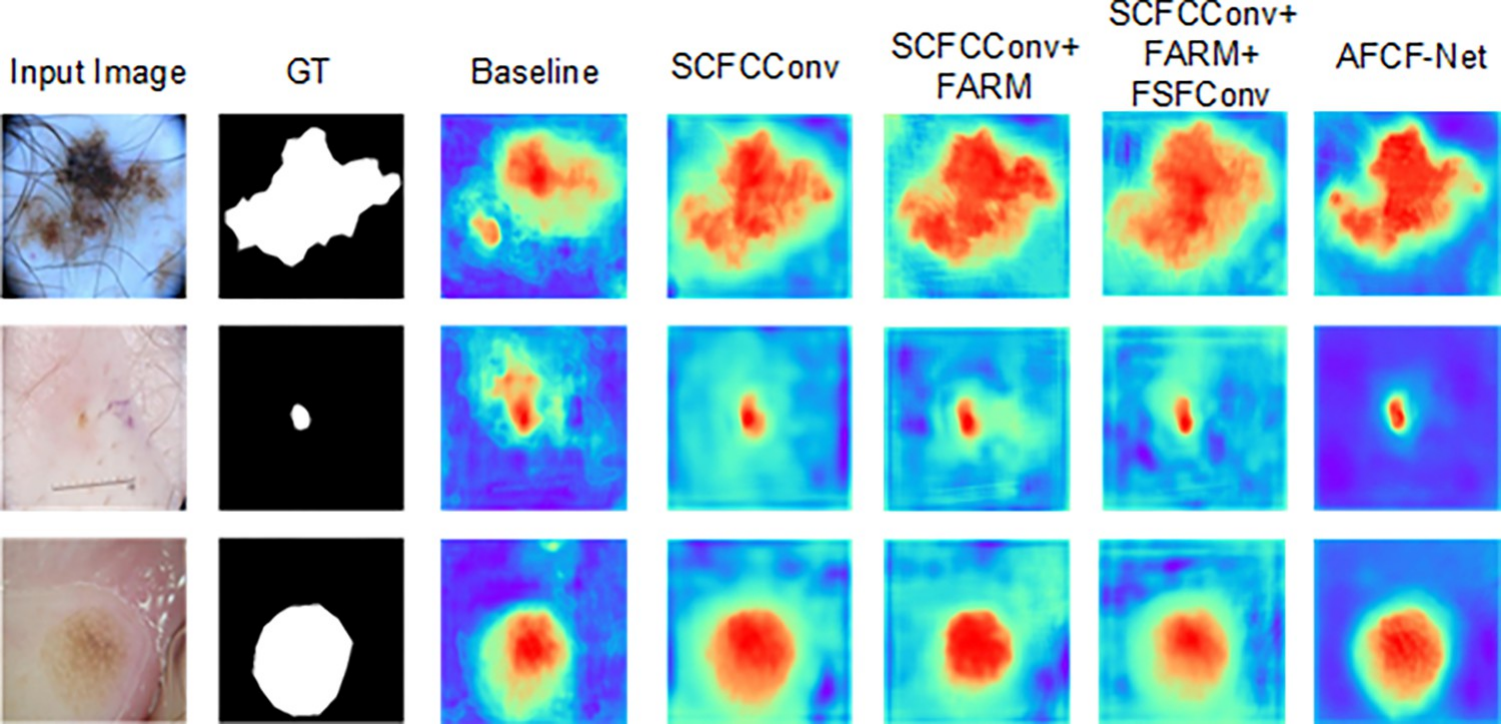
Attention heat maps of the ablation study experiments.

Fig 15 displays the attention heatmaps of the proposed AFCF-Net network after sequentially adding SCFCConv, FARM, FSFConv, and the multi-level feature aggregation branch to the baseline (warmer colors indicate higher attention coefficients). By comparing the heatmaps, it can be observed that as more components were added, the network gradually increased its attention to the lesion areas. This indicates that the four newly elaborated components effectively enhance the network’s focus on lesion targets, enabling it to locate and segment the lesion areas more accurately.

## 5 Discussion

Accurately understanding and segmenting different lesion structures contained in medical images is crucial for the prompt diagnosis and early treatment of skin diseases. Through semantic segmentation techniques, each pixel in an image can be assigned to its corresponding class or structure, thereby accurately identifying and segmenting lesion areas in the image. The proposed AFCF-Net adopts the newly designed SCFCConv to adaptively adjust the response of convolutional kernels, thereby achieving more accurate extraction and representation of lesion features of diverse types. In order to better verify the features, we explored the influence of different dilation rates of the atrous convolution in SCFCConv on the AFCF-Net segmentation performance. From the obtained experimental results, shown in Table 7, it can be observed that as the dilation rate increases, the segmentation performance of the proposed network also gradually improves. This indicates that larger dilation rates help the network better capture contextual information in images, thereby improving its ability to recognize complex structures and subtle features. However, using convolutions with larger dilation rates can cause incomplete sampling at the image boundaries (the segmentation performance of the network starts to decline when the dilation rate reaches 9), thereby affecting the accuracy of the segmentation results at the boundaries, which warrants further exploration in future research. To ensure that AFCF-Net achieves the best segmentation performance, we ultimately decided to use an atrous convolution with a dilation rate of 7 to SCFCConv.

**Table 7:** AFCF-Net segmentation performance results using different dilation rates of atrous convolution in SCFCConv.

Dilation rate	IoU (%)	DSC (%)	Acc (%)	Sen (%)
R = 1	82.83	89.50	94.68	89.90
R = 3	83.06	89.82	94.85	90.44
R = 5	83.13	89.78	94.79	90.96
R = 7 (*used*)	**83.55**	**90.24**	**95.06**	91.03
R = 9	83.21	89.85	94.83	**91.61**

Furthermore, we explored the impact of different dilation rate combinations in FARM on the segmentation performance of AFCF-Net. Smaller dilation rate combinations can enhance the segmentation accuracy of subtle lesions, while larger dilation rate combinations are helpful for handling larger lesions or segmenting the overall area. Reasonably selecting dilation rate combinations can balance the network’s utilization of local and global information. From the obtained experimental results, shown in Table 8, it can be observed that among the five dilation rate combinations, (2, 4, 6) achieves the best segmentation performance, according to the two main evaluation metrics (IoU and DSC). This may be because this combination covers small, medium, and large dilation rates, effectively capturing feature information at different scales. This ability to comprehensively utilize information at different scales makes the (2, 4, 6) combination performing best in skin lesion segmentation tasks.

**Table 8:** AFCF-Net segmentation performance results using different dilation rate combinations in FARM.

Dilation rate combinations	IoU (%)	DSC (%)	Acc (%)	Sen (%)
(1, 2, 3)	83.14	89.83	**95.08**	89.55
(2, 4, 6) (*used*)	**83.55**	**90.24**	95.06	91.03
(2, 6, 12)	83.12	89.89	94.89	90.79
(3, 6, 9)	83.09	89.61	94.76	90.15
(6, 12, 18)	83.37	90.00	94.90	**91.16**

**Fig 16:**
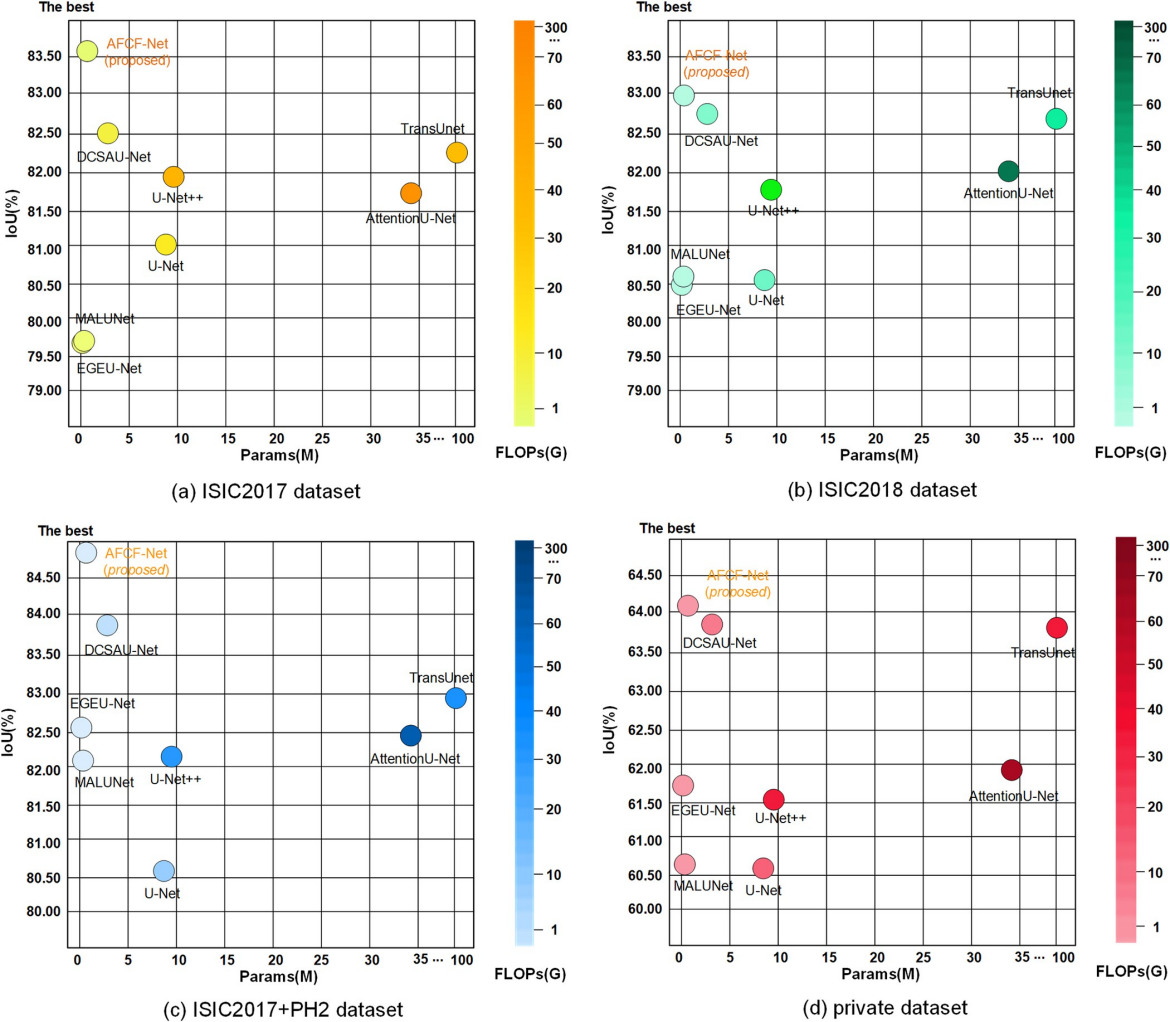
Network comparison in terms of the number of parameters, FLOPS, and IoU on different datasets (the closer the point of a network to the upper left corner, the better overall network performance; the lighter the color, the lower the FLOPS of a network).

Additionally, by visualizing the parameter counts, FLOPS, and IoU in Fig 16, one can more clearly observe the performance of the compared networks across the four datasets used. Large networks, like TransUNet and DCSAU-Net, excel in segmentation performance. Their outstanding performance highlights the potential of complex network architectures in handling fine-grained features and enhancing the segmentation performance. However, this performance improvement comes at the cost of increased computational expense, making it unsuitable for real-time applications with strict constraints on inference speed and computational resources. Lightweight networks, like MALUNet and EGE-UNet, can provide relatively stable performance with lower computational resources and faster inference speeds, but their segmentation performance may fall short in complex tasks compared to larger networks. In comparison, the proposed network shows the best performance across all four datasets, surpassing large networks in segmentation performance while also being on par with lightweight networks in terms of parameter count and FLOPS. However, although AFCF-Net already has fewer parameters and lower computational complexity, there is still potential for further optimization. In the future, we will continue to research lightweight modules and networks to further reduce the inference time of AFCF-Net.

## 6 Conclusion

A novel asymmetric encoder-decoder network, AFCF-Net, have been proposed in this paper for skin lesion segmentation. Four newly designed components were cleverly incorporated into the proposed network to enhance its segmentation performance. Firstly, a new convolution type, namely SCFCConv, was integrated into the U-Net network in order to calibrate and integrate feature information at different resolutions and receptive fields, allowing the network to adapt to different types of lesion features more flexibly. Secondly, another novel type of convolution, namely FSFConv, was employed at the skip connection to enhance the expressive capability of high-frequency features, enabling the network to focus more on changes in detailed features. Thirdly, introducing a newly designed FARM into the bottleneck allowed the network to fully utilize feature information at different scales outputted by the encoder, enhancing its perception ability towards lesions of different scales. Finally, a multi-level feature aggregation branch was introduced into the network to form a dual-decoder structure, providing additional feature representations to the original decoder. Additionally, a combined BCE-Dice loss was utilized to improve the network’s handling capability of imbalanced classes. Experimental results, obtained on the ISIC2017 and ISIC2018 public datasets, demonstrate that the proposed AFCF-Net can achieve state-of-the-art segmentation performance with significantly reduced resource demands. In another two sets of experiments, it was demonstrated that compared to other mainstream networks, AFCF-Net can handle more complex image features and has a stronger generalization ability.

Future research plans include reducing the number of parameters and computational complexity of AFCF-Net as to further improve its efficiency. Additionally, we will focus on enhancing the robustness of the network to make it applicable to a wider range of domains.
